# Composition of Microbiota in Transient and Mature Human Milk: Significant Changes in Large for Gestational Age Group

**DOI:** 10.3390/nu16020208

**Published:** 2024-01-09

**Authors:** Meltem Dinleyici, Vicente Pérez-Brocal, Sertac Arslanoglu, Ozge Aydemir, Sibel Sevuk Ozumut, Neslihan Tekin, Yvan Vandenplas, Andrés Moya, Ener Cagri Dinleyici

**Affiliations:** 1 Department of Social Pediatrics, Faculty of Medicine, Eskisehir Osmangazi University, 26480 Eskisehir, Türkiye; meltemayata@hotmail.com; 2 Department of Genomics and Health, Foundation for the Promotion of Health and Biomedical Research of Valencia Region (FISABIO-Public Health), 46020 Valencia, Spain; vicente.perez@fisabio.es (V.P.-B.);; 3 CIBER in Epidemiology and Public Health (CIBEResp), 28029 Madrid, Spain; 4 Division of Neonatology, Department of Pediatrics, Faculty of Medicine, Medeniyet University, 34720 Istanbul, Türkiye; 5 Division of Neonatology, Department of Pediatrics, Faculty of Medicine, Eskisehir Osmangazi University, 26040 Eskisehir, Türkiye; drozgegenc@yahoo.com.tr (O.A.); tekinneslihan@yahoo.com (N.T.); 6 KidZ Health Castle, UZ Brussel, Vrije Unversiteit Brussel, 1090 Brussels, Belgium; 7 Institute for Integrative Systems Biology (I2SysBio), University of Valencia and Spanish National Research Council (CSIC-UVEG), 46980 Valencia, Spain; 8 Department of Pediatrics, Faculty of Medicine, Eskisehir Osmangazi University, 26040 Eskisehir, Türkiye

**Keywords:** human milk, breastfeeding, microbiome, LGA, transient human milk, mature human milk

## Abstract

The composition of the human milk (HM) microbiota and, consequently, the microorganisms that are passed on to the infant through breastfeeding, can be influenced by various factors such as the mother’s health and diet, gestational age, delivery mode, lactation stage, method of infant feeding, and geographical location. The aim of the Human Milk-Gest Study was to compare the microbiota of transient (postpartum 7–15 days) and mature HM (postpartum 45–90 days) of 44 mothers, and to investigate any potential changes associated with preterm birth, mode of delivery, and birth weight in relation to gestational age. The data were classified into five study groups: normal spontaneous delivery-term (NS-T) newborns, cesarean delivery-term (CS-T) newborns, preterm (PT) newborns (with a gestational age of less than 37 weeks), small for gestational age (SGA) newborns, and large for gestational age (LGA) newborns. An analysis of differential abundance was conducted using ANCOM-BC to compare the microbial genera between transient and mature HM samples as well as between other study groups. A significant difference was detected between HM samples at different sampling times and between the study groups (*p* < 0.01). In transient HM samples, *Ralstonia*, *Burkholderiaceae_uc*, and *Pelomonas* were significantly dominant in the LGA group compared to the NS-T, CS-T, PT, and SGA groups. In mature HM samples, *Burkholderiaceae_uc*, *Ralstonia*, *Pelomonas*, and *Klebsiella* were significantly dominant in the LGA group compared to the NS-T, CS-T, and PT groups, while *Ralstonia*, *Burkholderiaceae_uc*, and *Pelomonas* were significantly dominant in the LGA group compared to the SGA group. Differences were also detected between the transient and mature HM samples in the CS-T, PT, SGA, and LGA groups, but no differences occurred in the NS-T groups. In conclusion, we showed that *Ralstonia*, *Burkholderiaceae_uc*, and *Pelomonas* were significantly dominant in the LGA group in transient HM and continued in mature HM. The body mass index (BMI) of the mothers in the LGA group was not >30 at conception, however, the maternal BMI at birth and maternal weight gain during pregnancy were higher than in the other groups. The nutritional composition of HM is specifically designed to meet infant nutritional requirements during early life. Evaluating the effects of HM microbiota on infant microbiota composition and short- and long-term health effects in larger studies would be useful.

## 1. Introduction

Breastfeeding is often regarded as the gold standard in infant nutrition, as human milk (HM) is specifically designed to meet the rapid growth needs of newborns in a timely manner [1,2]. Aside from being the main source of nutrition for infants, HM also plays a crucial role in introducing beneficial microbes into the infant gut during early life [1,3]. The HM microbiota refers to the collection of bacteria, viruses, fungus, and archaea that exist within HM, which is now recognized as a living ecosystem [1,4,5]. In general, consensus is lacking regarding the definition of a healthy HM “core” microbiome. Previous HM microbiota studies identified *Staphylococcus* and *Streptococcus* as the most prevalent genera in HM, regardless of geographical location. Various anaerobic microbes, including *Bifidobacterium*, *Faecalibacterium*, and *Akkermansia*, were present in HM. Additional identified genera have included lactic acid bacteria (specifically *Lactobacillus*), bacteria that are present on the skin (such as *Cutibacterium* and *Corynebacterium*), and bacteria that are commonly found in the gut (such as *Acinetobacter*, *Bacteroides*, *Ralstonia*, *Enterobacter Blautia*, *Clostridium*, *Dorea*, *Enterococcus*, *Escherichia*, *Mucispirillum*, *Pseudomonas*, *Rothia*, *Salmonella*, *Serratia*, *Shigella*, and *Veillonella*) [4,5]. Three distinct ideas have been suggested to explain the mechanisms by which these microorganisms are present in HM: the entero-mammary pathway, the retrograde inoculation pathway, and the concept of resident mammary microbiota [4].

The HM microbiota is subject to constant change and is affected by various factors such as the mother’s health and nutritional status, the mode of delivery, the gestational age, the sex of the infant, the number of previous pregnancies, the stage of lactation, the method of feeding, the geographic location, and the method used to collect and analyze HM samples [4,5]. Variations have been reported in the HM microbiota as the milk progresses from colostrum to transitional (postpartum 7–15 days) and mature (postpartum 45–90 days) HM [6]. The microbiota present in colostrum demonstrates broad bacterial diversity, with the dominant bacteria mainly belonging to the genera *Weisella*, *Leuconostoc*, *Staphylococcus*, *Streptococcus*, and *Lactococcus*. A progressive increase in the relative quantities of *Bifidobacterium*, *Enterococcus*, *Veillonella*, *Leptotrichia*, *Prevotella*, *Lactobacillus*, and *Staphylococcus* was then seen when comparing transitional and mature HM to colostrum [7]. Cabrero Rubio et al. [6] observed a gradual increase in the prevalence of common oral bacteria (such as *Veillonella*, *Leptotrichia*, and *Prevotella* spp.) in the transitional and mature HM. They also found higher levels of *Bifidobacterium* throughout the later phases of breastfeeding. Various other maternal factors such as breast diseases like mastitis, the mode of nursing (pumped or directly breastfed), intrapartum antibiotic use, and probiotic consumption might influence the composition of the HM microbiota [1,4,5,8,9,10]. Our previous investigation of the HM virome and mycobiome revealed changes in relation to the lactational stage, mode of delivery, and gestational age [11,12].

Breastfeeding and the composition of the HM bacterial microbiota may significantly affect the development of the infant gut microbiome [1,4,5]. Knowledge regarding the HM microbiota composition varies and is scarce regarding birth weight for gestational age. Therefore, the objective of the present study was to assess the composition of HM bacteria by conducting a comprehensive metagenomic analysis. A further goal was to compare the bacterial composition of transient and mature HM and to investigate any potential alterations associated with mode of delivery, preterm birth, and birth weight for gestational age.

## 2. Materials and Methods

The Human Milk-Gest Study is a prospective study performed in two university hospitals in Turkey to evaluate HM composition, including bacteria, viruses, fungi, and archaea [11,12]. The study design, inclusion criteria, and exclusion criteria have been previously reported [11]. A total of 88 HM samples were obtained from 44 mothers. Maternal age, mode of delivery, gestational age, birth weight, gender, and HM sampling time of the entire study group and subgroups are shown in Supplementary Table S1. There were five study groups: normal spontaneous delivery-term (NS-T) newborns, cesarean delivery-term (CS-T) newborns, premature (PT) newborns (gestational age < 37 weeks), small for gestational age (SGA) newborns, and large for gestational age (LGA) newborns. Multiple pregnancies, maternal age < 18 years or >45 years, maternal body mass index (BMI) > 30 kg/m^2^, use of probiotics and antibiotics during pregnancy or lactation, intrapartum antibiotic prophylaxis, and maternal gastrointestinal system disorder or psychiatric disorders were all criteria for exclusion from the study group. Mothers were classified according to their pre-gestational BMI as normal weight (BMI 18.5–24.9 kg/m^2^) and overweight (BMI ≥ 25.0–29.9 kg/m^2^). None of the mothers had a BMI > 30 at the time of conception, and none had a history of diabetes or other metabolic disorders. Three of the seven mothers developed gestational diabetes in LGA group.

Each mother provided their informed consent, including signing consent form permission for the collection of HM samples and subsequent analysis. HM samples were collected from mothers who were recruited from two university hospitals at two time points: transient HM samples (postpartum 7–15 days) and mature HM samples (postpartum 45–90 days). The HM samples were collected in the morning at the hospital and consisted exclusively of foremilk. Mothers were instructed to cleanse their nipples and the surrounding areas with sterile saline solution prior to collecting 3–5 mL of HM in sterile tubes. All HM samples were collected by manual hand expression. Prior to DNA extraction, all samples were stored at −20 °C. Detailed DNA extraction and sequencing have been previously reported [8].

The output files, consisting of raw sequencing reads in fastq format, were initially quality-filtered using the prinseq-lite program [13] and applying the following parameters: min_length: 50, trim_qual_right: 30, trim_qual_type: mean and trim_qual_window: 20. Forward and reverse reads passing the quality check were joined using the FLASH- 1.2.11 program [14] and applying the following parameters: min-overlap 10, max-overlap 150, and max-mismatch-density 0.1. Next, pairs, unpaired, and singleton reads were combined in unique files per sample. Then, to filter reads of human origin, the concatenated reads were mapped against the human genome (GRCh38.p11, reference human genome, December 2013) using bowtie2-2.3.4.1 with end-to-end and very sensitive options [15].

For taxonomic annotation, human-free sequences were concatenated as a unique file for taxonomy assignment, implemented with Kaiju v1.6.2 [16], and the resulting file was split by sample. Lineage information was added, and lineage names were parsed for undetermined ranks, counts of taxa abundance, and construction of an abundance matrix for all samples using the free statistical package R 3.1.0 [17]. In parallel, functional annotation analyses were carried out on the human-free sequences obtained after bowtie2 filtering. First, the reads were assembled for each sample using MEGAHIT v1.1.2 [18] and then aligned to the resulting contigs to identify which reads had assembled. Those that did not assemble were appended to the contigs for use with the gene prediction program Prodigal v2.6.3 [19]. Functional annotation was carried out with HMMER [20] against the bacteria non-supervised orthologous groups (bactNOG) protein database of eggNOG 4.5 [21]. The reads to the open reading frames (ORF) were aligned using megablast, and the filtering of the best annotations and the assignment of the ORF annotation to every read were carried out using the package R. The R package was also used to count the aligned reads, to add the category and its coverage, and, finally, to build contingency tables. The resulting taxonomic and functional abundance matrices were converted into the biom format for abundance and diversity analyses using QIIME pipeline version 1.9.0 [22].

For taxonomy, alpha diversity or diversity within sample analysis was determined with 1000 replicates of randomly chosen subsets of 1000 reads per sample, with replacement, and the Shannon diversity index was calculated for those 1000 replicates. Boxplots were created using the R statistical package and statistically significant differences were determined using two-tailed *t*-tests. Statistical analysis was conducted using the R 3.1.0 software packages [17].

Differential abundance analyses of the species and genera were performed using ANCOM-BC with false discovery rate (FDR) correction [23]. Paired comparisons were made between the transient and mature HM samples, and unpaired comparisons were made between the HM samples based on delivery mode, birth weight, and gestational week. The variables in the normalized ANCOM-BC data were subjected to signal filtering, and the recalculated adjusted *p*-values were obtained. Subsequently, taxa that exhibited adjusted *p*-values below 0.05 were graphically represented as volcano plots for each comparison pair at the genus level. This was accomplished using the VolcaNoseR web application [24], with the log2 fold change also included in the plots.

In terms of taxonomic and functional analyses, beta diversity, which measures the dissimilarity between samples, was calculated using the Bray–Curtis dissimilarity index matrices and shown as principal coordinates analysis (PCoA) [17]. The nonparametric statistical method for multivariate analysis of variance, Adonis [25], was used to compare categories in both matrix files. Further analyses such as Wilcox tests were conducted using R scripts.

The metagenome data sets from this study can be found in the EBI Short Read Archive. They are associated with the study accession number PRJEB26810 and have individual accession numbers ranging from ERS2488898 to ERS248888985.

## 3. Results

### 3.1. Alpha Diversity

**Figure 1:**
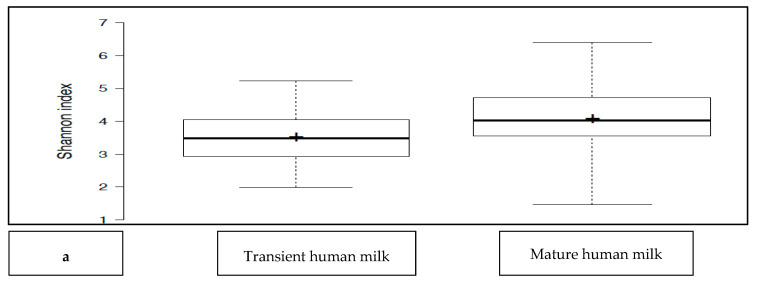

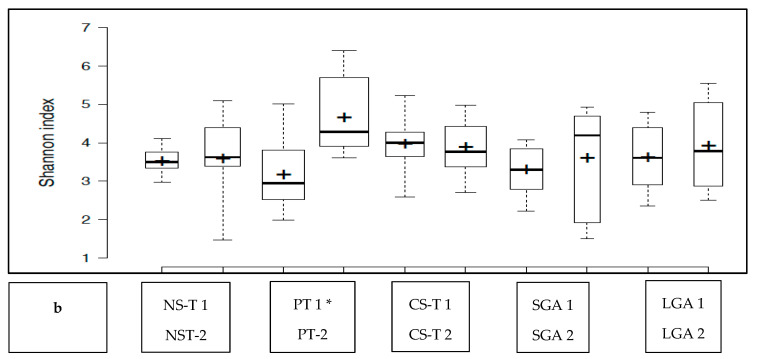
(**a**): Comparison of the median Shannon index values between transient human milk (HM) and mature HM. (**b**): Comparison of the median Shannon index values between transient HM and mature HM in the study groups. NS-T: normal spontaneous vaginal delivery-term; CS-T: cesarean delivery-term; PT: premature; SGA: small for gestational age; LGA: large for gestational age; SV: spontaneous vaginally; CS: cesarean delivery. 1: Transient human milk. 2: Mature human milk. * *p* < 0.05 for Shannon index between transient human milk vs. mature human milk.

The transient HM and the mature HM had similar Shannon index values (a measure of richness and uniformity that considers entropy), indicating similar alpha diversity between the groups (*p* > 0.05) (Figure 1a). No difference was noted between the study groups at two different time points (*p* > 0.05 for all). The exception was the premature group, which showed a lower median Shannon index for the transient HM than for the mature HM (*p* < 0.05) (Figure 1b).

### 3.2. Human Milk Microbiota Composition

**Figure 2:**
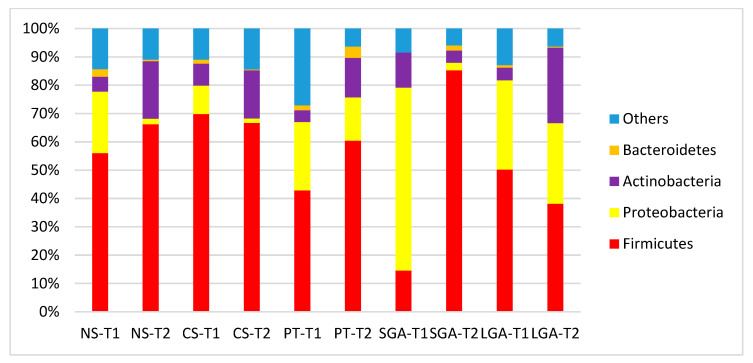
Comparison of human milk bacteria composition at the phylum level. NS-T: normal spontaneous vaginal delivery-term; CS-T: cesarean delivery-term; PT: premature; SGA: small for gestational age; LGA: large for gestational age; T1: Transient human milk. T2: Mature human milk.

**Figure 3:**
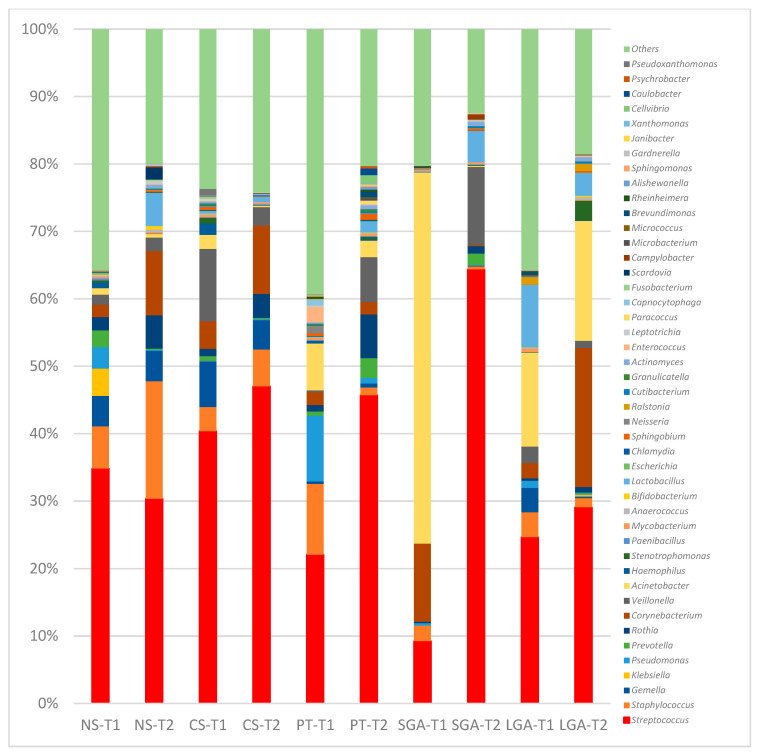
Comparison of human milk bacteria composition at the genus level. NS-T: normal spontaneous vaginal delivery-term; CS-T: cesarean delivery-term; PT: premature; SGA: small for gestational age; LGA: large for gestational age; T1: Transient human milk. T2: Mature human milk.

The comparisons of HM microbiota composition at the phylum and genus levels and the mode of delivery, gestational age, and birth weight at two different sampling times (transient and mature HM) are shown in Figure 2 and Figure 3. In the transient HM samples, Proteobacteria were the most abundant at the phylum level in the NS-T and SGA groups, whereas Firmicutes were the most abundant phylum in the CS-T group. In mature HM samples, Firmicutes were the most abundant phylum in all groups.

At the genus level, in the NS-T group, *Enterobacterales_uc*, *Klebsiella*, and *Pseudomonas* were the most abundant genera in transient HM samples, while *Streptococcus* and *Corynebacterium* were the most abundant in mature HM samples. In the CS-term group, *Streptococcus* was the most abundant genus in transient and mature HM samples. In the premature group, *Pseudomonas* was the most abundant genus in the transient HM samples, and different genera were observed when compared with the other groups. In the mature HM samples, *Streptococcus* was the most abundant genus. In the SGA group, *Acinetobacter* was the most abundant genus in transient HM samples, whereas *Streptococcus* was the most abundant in mature HM samples. In the LGA group, *Acinetobacter* and *Streptococcus* were the most abundant genera in both the transient and mature HM samples.

### 3.3. Differential Abundance Analysis of the Genera between Study Groups

**Figure 4:**
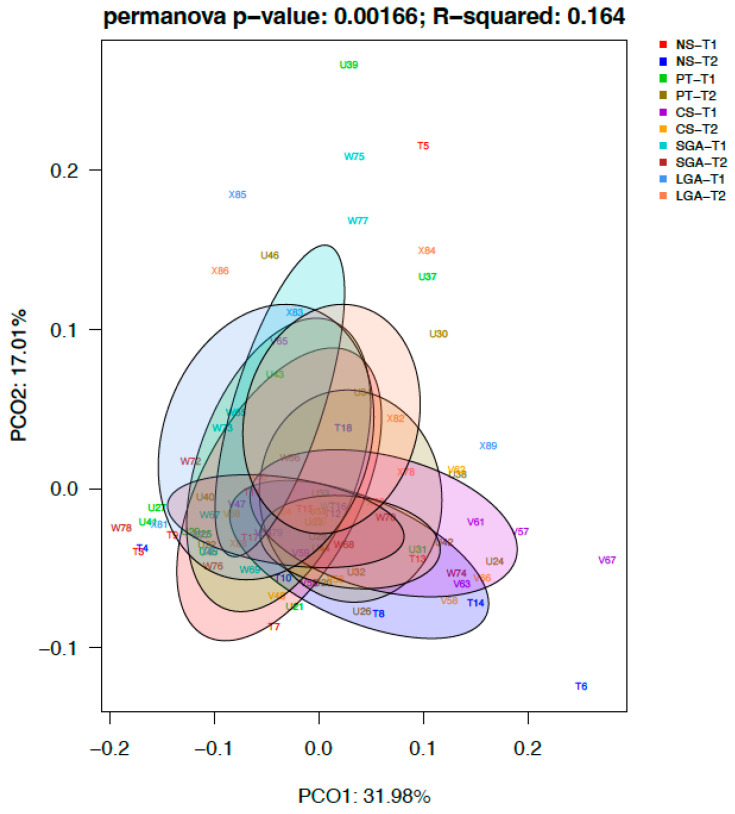
Difference between HM samples at different sampling times and between the study groups (*p* < 0.01). NS-T: normal spontaneous vaginal delivery-term; CS-T: cesarean delivery-term; PT: premature; SGA: small for gestational age; LGA: large for gestational age; T1: Transient human milk. T2: Mature human milk.

The differential abundance of the genera was analyzed using ANCOM-BC for paired comparisons between the transient and mature HM samples and unpaired comparisons between HM samples between the NS-T, CS-T, PT, SGA, and LGA groups at two different sampling times. A difference was detected between HM samples collected at different sampling times and between the study groups (*p* < 0.01) (Figure 4). The volcano plot graphics show the bacterial taxa that were significantly different in abundance between the study groups and in milk collected at different sampling times.

### 3.4. Transient HM Samples

**Figure 5:**
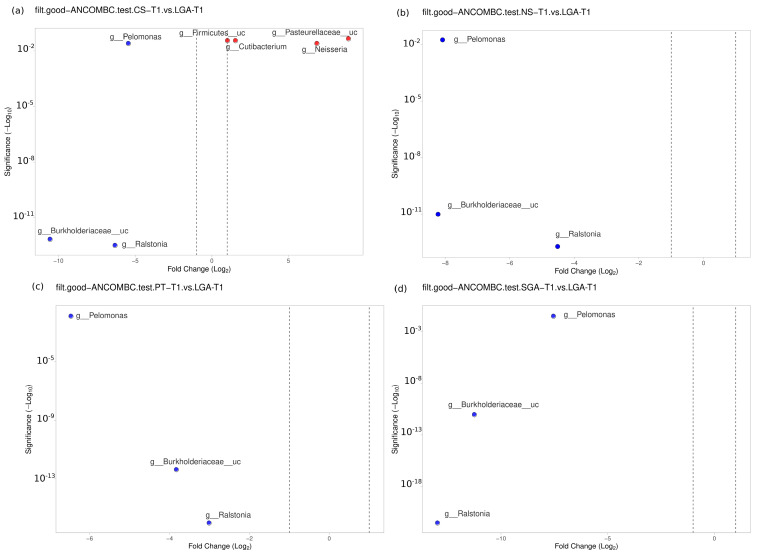
Differential abundance analysis of the genera was carried out using ANCOM-BC for unpaired comparisons between HM samples between the LGA and CS-T, NS-T, PT, and SGA groups in transient HM samples. Blue dots: most significantly overrepresented genera in the LGA group. Red dots: most significantly overrepresented genera in other groups. NS-T: normal spontaneous vaginal delivery-term; CS-T: cesarean delivery-term; PT: premature; SGA: small for gestational age; LGA: large for gestational age; T1: Transient human milk.

No difference was noted between the NS-T group and CS-T group or the PT group in the transient HM samples. At the genus level, *Bacillus* was found to be dominant in the SGA group compared to the NS-T group (*p* < 0.05). *Ralstonia* (*p* < 0.001), *Burkholderiaceae_uc* (*p* < 0.001), and *Pelomonas* (*p* < 0.01) were also significantly dominant in the LGA group compared to the NS-T group (Figure 5).

A difference was detected between the CS-T group and the PT group (*p* < 0.05). At the genus level, *Streptococcus* (*p* < 0.05), *Selonomonas* (*p* < 0.05), and *Peptoniphilus* (*p* < 0.01) were found to be significantly dominant in the CS-T group compared to the PT group. In the CS-T group, *Peptoniphilus* (*p* < 0.001), *Streptococcaceae_uc* (*p* < 0.01), *Lactobacillales_uc* (*p* < 0.01), *Haemophilus* (*p* < 0.01), and *Selenomonas* (*p* < 0.01) were dominant compared to the SGA group. A significant difference was detected between the CS-T group and the LGA group (*p* < 0.04). While *Neisseria* (*p* < 0.01), *Cutibacterium* (*p* < 0.05), *Firmicutes_uc* (*p* < 0.01), and *Pasteurellaceae_uc* (*p* < 0.01) were dominant in the CS-T group, *Ralstonia* (*p* < 0.001), *Burkholderiaceae_uc* (*p* < 0.001), and *Pelomonas* (*p* < 0.01) were significantly dominant in the LGA group. *Ralstonia* (*p* < 0.001), *Burkholderiaceae_uc* (*p* < 0.001), and *Pelomonas* (*p* < 0.01) were also significantly dominant in the LGA group compared to the PT group. No differences were noted between the PT group and the SGA group (Figure 5).

### 3.5. Mature HM Samples

**Figure 6:**
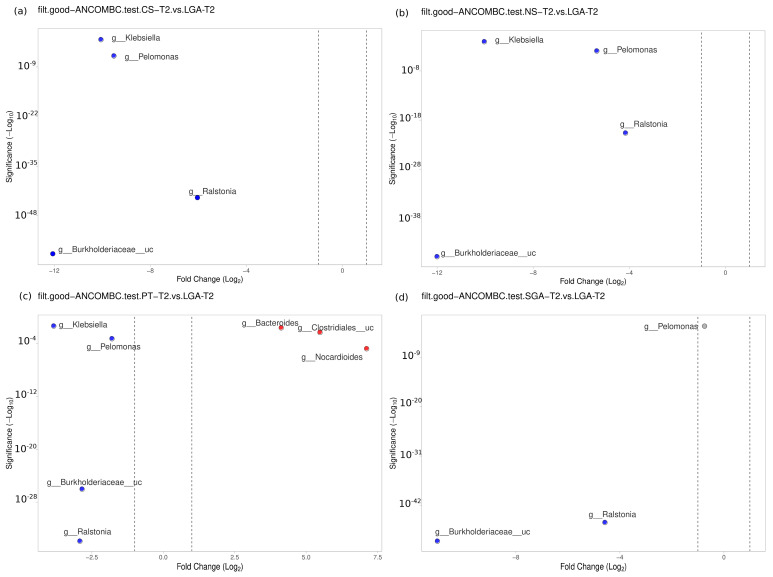
Differential abundance analysis of the genera was carried out using ANCOM-BC normalized data for unpaired comparisons between HM samples between the LGA and CS-T, NS-T, PT, and SGA groups in mature HM samples. Blue dots: most significantly overrepresented genera in the LGA group. Red dots: most significantly overrepresented genera in other groups. NS-T: normal spontaneous vaginal delivery-term; CS-T: cesarean delivery-term; PT: premature; SGA: small for gestational age; LGA: large for gestational age. T2: Mature human milk.

No difference was detected between the NS-T and CS-T groups. *Moraxella* was dominant in the PT group compared to the NS-T group (*p* < 0.05). *Micrococcus* was dominant in the NS-T group compared to the SGA group (*p* < 0.05). *Burkholderiaceae_uc* (*p* < 0.001), *Ralstonia* (*p* < 0.001), *Pelomonas* (*p* < 0.001), and *Klebsiella* (*p* < 0.01) were also significantly dominant in the LGA group compared to the NS-T group (Figure 6).

A difference was evident between the CS-T and PT groups. At the genus level, *Bacteroides* was dominant in the PT group compared to the CS-T group (*p* < 0.05). In the CS-T group, *Bacillales_uc* (*p* < 0.001), *Gemella* (*p* < 0.001), and *Corynebacteriaceae_uc* (*p* < 0.01) were significantly dominant compared to the SGA group, while *Burkholderiaceae_uc* (*p* < 0.001), *Ralstonia* (*p* < 0.001), *Pelomonas* (*p* < 0.001), and *Klebsiella* (*p* < 0.05) were significantly dominant in the LGA group compared to the CS-T group (Figure 6).

No difference was found between the PT and SGA groups. Again, *Burkholderiaceae_uc* (*p* < 0.001), *Ralstonia* (*p* < 0.001), *Pelomonas* (*p* < 0.001), and *Klebsiella* (*p* < 0.01) were significantly dominant in the LGA group compared to the PT group, *Nocardiodes* (*p* < 0.001), *Clostridiales_uc* (*p* < 0.001), and *Bacteroides* (*p* < 0.01) were dominant in the PT group. *Ralstonia* (*p* < 0.001), *Burkholderiaceae_uc* (*p* < 0.001), and *Pelomonas* (*p* < 0.05) were significantly dominant in the LGA group compared to the SGA group (Figure 6).

### 3.6. Comparison between Transient and Mature HM Samples

**Figure 7:**
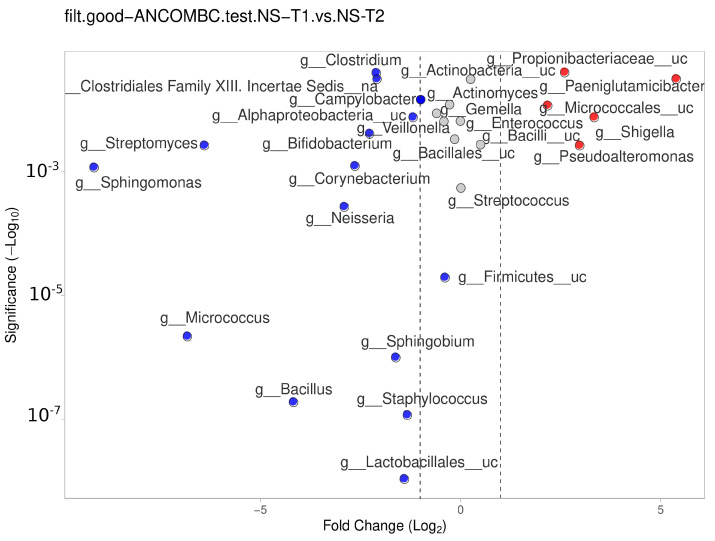
Differential abundance analysis of the genera was carried out using ANCOM-BC normalized data for paired comparisons between the HM samples of the NS-T group between transient and mature HM samples. Red dots: most significantly overrepresented genera in transient HM samples. Blue dots: most significantly overrepresented genera in mature HM samples. NS-T: normal spontaneous vaginal delivery-termç T1: Transient human milk. T2: Mature human milk.

The NS-T group showed a significant difference between the transient and mature HM samples. In mature HM milk samples, *Lactobacillales__uc*, *Staphylococcus*, *Bacillus*, *Sphingobium*, *Micrococcus*, *Firmicutes__uc*, *Neisseria*, *Sphingomonas*, *Corynebacterium*, and other genera in Figure 7 (blue dots) were more represented in the NS-T group.

In the CS-T group, *Neisseria* (*p* < 0.001), *Pasteurellaceae_uc* (*p* < 0.01), *Selenomonas* (*p* < 0.01), and *Escherichia* (*p* < 0.01) were dominant in transient HM samples compared to mature HM samples. In the PT group, while the *Salmonella* (*p* < 0.01) genus was dominant in transient HM samples, more than 20 genera were abundant in mature HM samples. In the SGA group, *Bacillus* (*p* < 0.001), *Proteobacteria_uc* (*p* < 0.001), *Escherichia* (*p* < 0.01), *Pseudomonadales_uc* (*p* < 0.01), and *Acinetobacter* (*p* < 0.01) predominated in transient HM, while *Prevotella* (*p* < 0.001), and *Veillonella* (*p* < 0.01) were abundant in mature HM. In the LGA group, *Paenibacillus* (*p* < 0.001), *Salmonella* (*p* < 0.001), and *Mycobacterium* (*p* < 0.001) were significantly dominant in transient HM, while *Bifidobacterium* (*p* < 0.001), *Klebsiella* (*p* < 0.01), and *Stenethropomonas* (*p* < 0.01) were predominate in mature HM.

Regarding the difference between the groups at species levels, in transient HM samples, *Shigella sonnei* (*p* < 0.01) and *Bordatella bronchisepticum* (*p* < 0.05) were found to dominant in NS-T group comparing the CS-T, while *Streptococcus* sp. *S4643* (*p* < 0.05) is dominant in CS-T group. In NS-T group, *Leuconostoc* sp. *DORA_2* (*p* < 0.01) and *Shigella sonnei* (*p* < 0.01) is dominant comparing the PT group. *Ralstonia mannitolytica* (*p* < 0.05) is dominant in the LGA group comparing NS-T group. While delivery mode is *C*-section for both CS-T and premature group, *Streptococcus salivarius*, *Streptococcus* sp. *ACS2*, *Veillonella parvula*, *Veillonella SP DNF0669*, *Cutibacterium acnes*, *Streptococcus pyogenes,* and *Streptococcus* sp. *SK 643* are significantly dominant in CS-T group rather than PT group (*p* < 0.05 for all). In CS-T group, *Streptococcus salivarius*, *Streptococcus vestibularis, and Cutibacterium acnes* is dominant than SGA group, and *Cutibacterium acnes* is dominant than LGA group (*p* < 0.05 for all). Between SGA and LGA group, *Ralstonis mannitolycia* and *Ralstonia_uc* were dominant in LGA group (*p* < 0.05 for all).

Regarding the difference between the groups at species levels, in mature HM samples, *Streptococcus suis* (*p* < 0.01) and *Streptococcus* sp. *HS1503* (*p* < 0.05) were found to dominate in NS-T group comparing the CS-T, while *Streptococcus* sp. *S4643* (*p* < 0.05) is dominant in CS-T group. *Proteobacteria_uc*, *Leuconostoc* sp. *DORA_2*, *Streptococcus australis,* and *Streptococcus equi* is dominant in PT comparing the NS-T group (*p* < 0.05). *Streptococcus anginosus* and *Veillonella dispar* is dominant in NS-T group comparing the SGA group (*p* < 0.05 for both). *Streptococcus* sp. *263_SSPC* is dominant in NS-T group, comparing the LGA group. There is no statistically significant difference between CS-T and PT group. *Rothia mucilaginosa* is dominant in PT group comparing the SGA group (*p* < 0.05).

No significant differences were identified between the groups for functional enrichment with bacteria non-supervised orthologous groups (bactNOG) functional categories (S). We determined some functional metabolic pathway differences between the groups. The transient HM samples showed significant differences in bacterial metabolic pathways between the CS-T group and the PT group. In transient HM samples, the CS-T group showed a higher abundance of bacterial metabolic pathways associated with nucleotide transport and metabolism (adenylsuccinate lyase) compared to the SGA group (*p* < 0.05).

## 4. Discussion

Variations have been reported in the HM microbiota as the milk progresses from colostrum to transitional and mature HM [6,26]. In our study, the NS-T group showed significant differences between the transient and mature HM samples. Gonzalez et al. [27] discovered that HM samples obtained during the initial phase of lactation (6–46 days after giving birth) contained a high abundance of *Staphylococcus* and *Streptococcus* spp., which were associated with the oral and intestinal tracts of infants. Conversely, HM samples collected during the later stage (109–184 days post-partum) were dominated by other species such as *Sphingobium* and *Pseudomonas*, which are involved in the degradation of aromatic compounds [27]. We also observed significant changes in the genera comprising the microbiota composition in the PT group, CS-T group, SGA group, and in the LGA group. The changes we observed between transient and mature HM samples clearly confirmed that the HM microbiota composition changes over time and reflects the characteristics of the infant. Variations have been reported in the HM microbiota as the milk progresses from colostrum to transitional and mature HM [6]. This observed change throughout the lactation period can be partially attributable to the retrograde inoculation pathway, as an increased amount of normal oral bacteria has been documented in HM samples collected during the later stages of lactation. The species that were more abundant during the early lactation period consisted of potential commensal bacteria known to inhabit the oral and intestinal tracts of infants. By contrast, the species that were more abundant during the late lactation period shared a common functional feature related to the breakdown of aromatic compounds [7].

Our study is the first to show the unique changes occurring in the HM microbiota composition in the LGA group. The occurrence of a newborn with a larger size than expected for their gestational age, known as an LGA phenotype, is primarily associated with parental gestational or pre-existing diabetes, insulin resistance, and/or obesity [28]. In transient HM samples, *Ralstonia*, *Burkholderiaceae_uc*, and *Pelomonas* were significantly dominant in the LGA group compared to the NS-T, CS-T, PT, and SGA groups. The mature HM samples also showed dominance of *Burkholderiaceae_uc*, *Ralstonia*, *Pelomonas*, and *Klebsiella* in the LGA group compared to the NS-T, CS-T, and PT groups, and *Ralstonia*, *Burkholderiaceae_uc*, and *Pelomonas* were also significantly dominant in the LGA group compared to the SGA group. When we checked the HM microbiota composition of the *Ralstonia* genera at the species level, the most frequently observed species were *Ralstonia mannitolilytica*, *Ralstonia pickettii*, and *Ralstonia* sp. *UNCCL144*.

In the LGA group, we found an increase in similar bacterial genera in both transitional and mature HM, independent of the mode of delivery and gestational week, suggesting a direct effect of LGA birth-related conditions on the HM microbiota in these cases. All the LGA babies were born by *C*-section. None of the mothers had a BMI >30 at the time of conception, and none had a history of diabetes or other metabolic disorders. However, the BMI of these mothers in the LGA group at the time of birth and their weight gain during pregnancy were higher than the values seen in the other groups, and three of the seven mothers developed gestational diabetes. Karlsson et al. [29] conducted a study to compare the composition of gut microbiota in a specific group of 10 LGA newborns who had been delivered vaginally without any complications at an average gestational age of 40 weeks, and had an average birth weight of 3682 g. Their study was the first to show that LGA newborns have a distinct microbiota composition in the first two days of life compared to newborns born with an average birth weight. The study focused specifically on newborns who had noncomplicated vaginal births. The neonates born LGA had a greater occurrence of gram-negative *Proteobacteria*, while neonates born AGA had a higher occurrence of gram-positive Firmicutes. The authors suggested that the altered microbiota in LGA infants may have reflected the fact that these newborns may have had mothers who were obese, as maternal weight is known to affect the composition of the infant’s microbiota [29].

Information about HM microbiota in LGA is quite limited. The occurrence of gestational difficulties is linked to a delay in the start of secretory activation in LGA babies, and is related to immature feeding patterns, dependence on breast pump usage, or possible anomalies in the mammary gland [28]. The presence of defective or delayed initiation of secretion, combined with an infant who has difficulty or is unable to suck effectively and efficiently shortly after birth, frequently leads to the inability to establish a successful nursing relationship, resulting in the need to supplement with formula either partially or completely [28]. HM from mothers with gestational diabetes and/or insulin resistance also shows metabolomic abnormalities, such as a decrease in the concentration of proteins involved in glucose homeostasis when compared to HM from mothers with normal pregnancies [28]. Cortes-Macias and colleagues [30] also evaluated the effect of mixed and exclusive breastfeeding practices on the HM microbiota and evaluated the effects of pre-pregnancy body mass index and weight gain over pregnancy on its composition. Contrary to our results, they found that normal-weight mothers had a higher prevalence of *Bifidobacterium* and *Ralstonia* and a lower incidence of *Staphylococcus* in HM compared to overweight mothers. They also showed that exclusively breastfeeding mothers displayed a greater incidence of *Ralstonia* in their HM compared to mixed-feeding mothers [30]. However, their study provided no information about the percentage of LGA babies in their study population.

*Ralstonia* is known to increase in the gut microbiota of obese adults with type 2 diabetes and reciprocally worsens glucose tolerance [31]. The gut microbiota alterations can impact the composition of HM and, subsequently, the development of the infant microbiome. Notable disparities in HM metabolite levels between mothers who are overweight or obese and mothers who have a normal weight have also been reported [4,32]. Whether newborns with LGA at birth, particularly when they exhibit abnormalities in appetite hormones and oral feeding capacity at birth [28], are at risk for development and metabolic complications or experience any unique effects of HM composition, including microbiota, remains an unanswered question. We found three bacterial genera, all Burkholderiales order members, in transient and mature HM samples in the LGA group, therefore, the specific mechanism that leads to the occurrence of these potentially novel microbiota biomarkers needs further evaluation.

Studies on the effect of the mode of delivery on the composition of HM microbiota have provided contradictory results [4,5,33]. In our study, we observed no difference in the richness or microbiota composition between the NS-T group and the CS-T group in either the transient HM samples or the mature HM samples. In our study, although almost all of the children in the PT, SGA, and LGA groups were born by cesarean section, differences were noted in terms of the HM microbiota in the CS-T group, suggesting that differences arise in microbiota through events other than mode of delivery. Although the indication for cesarean section in children in the PT, SGA, and LGA groups was frequently an emergency delivery, the indication for cesarean section in the CS-T group was elective, which could suggest that the perinatal reasons for the emergency cesarean section affected the HM microbiota composition. The effects of emergency and elective cesarean section on infant’s gut microbiota have been shown to differ [34].

Studies on the effect of the preterm birth on the composition of HM microbiota have provided contradictory results [5,34,35]. Urbaniak and colleagues [35] did not observe any alterations in the HM microbiota composition associated with gestational age, and suggested the existence of a fail-safe mechanism that enables the mother to transmit her bacterial imprint to the baby, irrespective of the infant’s gestational age at birth [35]. In our study, we observed some difference between the PT group and other study groups. The majority of the babies in the PT group were born by *C*-section, and we observed a difference in the transient and mature HM samples between the CS-T group and the PT group at the genus level. In preterm newborns, many environmental factors, prematurity-related conditions, and nutritional differences occur, and these same factors could affect the HM microbiota composition at the individual level.

This study is subject to limitations, primarily due to the small sample size of its subgroups, which diminishes the likelihood of identifying additional significant relationships. Maternal weight at conception, maternal weight gain during pregnancy, maternal obesity, gestational diabetes, maternal dietary preferences, and maternal stress might affect the HM microbiota analysis. Further studies including these maternal characteristics would help to understand microbiota composition. Regarding our study protocol, we did not evaluate the microbiota composition of colostrum, and this is a gap in our exploration of the HM microbiota trajectory during the early neonatal period. While we found significant changes in the LGA group, the size of that group is small, and LGA-born babies tend to be a heterogenous group in terms of pre- and peri-natal factors. Other HM components, such as HM oligosaccharides (HMOs), are also speculated to modulate the composition of HM microbiota [7,36]. Further studies that include the HMO profile and HM microbiota composition would help to understand these interactions, especially in the LGA group.

Regardless of its source, the HM microbiota can contribute to the overall well-being of mothers and their infants through various functions that support the healthy development of gut microbiota, inhibit the growth of harmful bacteria, and have a regulatory impact on metabolic pathways and immune responses. The developmental origins of health and disease hypothesis suggests that environmental exposures throughout early life can modify the programming of fetuses and infants, leading to changes in their health status [37]. One of the things that can be changed by these early-life exposures is the maintenance of the gut microbiota in infants, which is influenced by the composition of the HM microbiota. 

## 5. Conclusions

The HM composition undergoes changes based on maternal and neonatal circumstances, and these changes may be associated with adjustments of HM to meet the specific requirements of newborns. Conducting larger research studies will be beneficial in assessing the impact of the dominant bacterial species identified in the transitional HM of LGA newborns and present in mature HM on the composition of infant microbiota and the long-term health implications.

## Data Availability

The metagenome data sets from this study can be found in the EBI Short Read Archive. They are associated with the study accession number PRJEB26810 and have individual accession numbers ranging from ERS2488898 to ERS248888985.

## Supplementary Materials

The following supporting information can be downloaded at: https://www.mdpi.com/article/10.3390/nu16020208/s1, Supplementary Table S1. Maternal age, maternal weight status, mode of delivery, gestational age, birth weight, gender, and human milk sampling time of entire study group and subgroups.

## Abbreviations

Human milk (HM), normal spontaneous delivery-term (NS-T), cesarean delivery-term (CS-T), premature (PT), small for gestational age (SGA), large for gestational age (LGA), uncharacterized (__uc), no taxonomic assignment (n_ or _n), body mass index (BMI).
