# Supplementary material for: Composition of Microbiota in Transient and Mature Human Milk: Significant Changes in Large for Gestational Age Group
Source: Nutrients. 2024 Jan 9;16(2):208. doi: 10.3390/nu16020208 (PMC10818272; doi:10.3390/nu16020208)
Supplement: Supplementary file 1 [file nutrients-16-00208-s001.zip › nutrients-2798180-supplementary.pdf]

Supplementary Table S1. Maternal age, maternal weight status, mode of delivery, gestational age, birth weight, gender, and human milk sampling time of entire study group and subgroups.

	Maternal Age (years) *	Maternal weight status (NW/OW)	Delivery Mode (SV/CS)	Gestational Age (weeks) *	Birth Weight (gram) *	Gender (Boys/Girls)	Infant Feeding EBF/MF	Transient HM Sampling Time (days) *	Mature HM Sampling Time (days) *
Total (*n* = 44)	30 (18–44)	35/9	9/35	38 (32–41)	2840 (1200–4600)	18/26	27/17	8 (7–14)	51.5 (45–70)
NS-T (*n* = 8)	33 (20–41)	6/2	8/0	38.5 (37–40)	3225 (2880–3600)	1/7	5/3	12 (8–14)	53.5 (47–70)
CS-T (*n* = 9)	35 (25–44)	7/2	0/9	38 (37–41)	3080 (2730–3600)	3/6	6/3	8 (7–13)	55 (45–70)
PT (*n* = 13)	30 (18–43)	11/2	0/13	35 (32–37)	2255 (1200–2700)	5/8	6/7	8 (7–14)	50 (45–62)
SGA (*n* = 7)	34 (22–38)	6/1	1/6	37 (34–41)	2225 (1670–2500)	3/4	4/3	8 (7–12)	49 (45–63)
LGA (*n* = 7)	30 (23–39)	5/2	0/7	39 (37–41)	4120 (3505–4600)	6/1	6/1	8 (7–13)	51 (45–63)

* median (minimum–maximum). NS-T: normal spontaneous vaginal delivery-term; CS-T: cesarean delivery-term; PT: premature; SGA: small for gestational age; LGA: large for gestational age; SV: spontaneous vaginally; CS: cesarean delivery. Mothers were classified according to their pre-gestational BMI as normal weight (BMI 18.5–24.9 kg/m2) and overweight (BMI ≥25.0-29.9 kg/m2). EBF: exclusively breastfeeding MF (mixed feeding: breastfeeding and formula feeding).
